# Visual Cortex Transcranial Direct Current Stimulation for Proliferative Diabetic Retinopathy Patients: A Double-Blinded Randomized Exploratory Trial

**DOI:** 10.3390/brainsci11020270

**Published:** 2021-02-21

**Authors:** Angelito Braulio F. de Venecia, Shane M. Fresnoza

**Affiliations:** 1 Eye Center, Region 1 Medical Center, Dagupan 2400, Pangasinan, Philippines; abfdv777@gmail.com; 2 Nazareth General Hospital, Dagupan 2400, Pangasinan, Philippines; 3 Institute of Psychology, University of Graz, 8010 Graz, Austria; 4 BioTechMed, 8010 Graz, Austria; 5 College of Medicine, De La Salle Health Sciences Institute, Dasmarinas 4114, Cavite, Philippines

**Keywords:** transcranial direct current stimulation, visual acuity, numerical discrimination, diabetes, diabetic neuropathy, visual cortex

## Abstract

Proliferative diabetic retinopathy (PDR) is a severe complication of diabetes. PDR-related retinal hemorrhages often lead to severe vision loss. The main goals of management are to prevent visual impairment progression and improve residual vision. We explored the potential of transcranial direct current stimulation (tDCS) to enhance residual vision. tDCS applied to the primary visual cortex (V1) may improve visual input processing from PDR patients’ retinas. Eleven PDR patients received cathodal tDCS stimulation of V1 (1 mA for 10 min), and another eleven patients received sham stimulation (1 mA for 30 s). Visual acuity (logarithm of the minimum angle of resolution (LogMAR) scores) and number acuity (reaction times (RTs) and accuracy rates (ARs)) were measured before and immediately after stimulation. The LogMAR scores and the RTs of patients who received cathodal tDCS decreased significantly after stimulation. Cathodal tDCS has no significant effect on ARs. There were no significant changes in the LogMAR scores, RTs, and ARs of PDR patients who received sham stimulation. The results are compatible with our proposal that neuronal noise aggravates impaired visual function in PDR. The therapeutic effect indicates the potential of tDCS as a safe and effective vision rehabilitation tool for PDR patients.

## 1. Introduction

Diabetic retinopathy (DR) is a grave ocular complication of diabetes mellitus and the leading cause of preventable blindness. The incidence of DR is projected to increase because the number of diabetic patients is expected to rise from 171 million in 2000 to 366 million by 2030 worldwide [1]. The economic burden of DR comes from direct disease management costs and lost worker productivity because it affects working-age populations [2]. Clinically, in the early asymptomatic stage of DR (non-proliferative diabetic retinopathy (NPDR)), microaneurysms, hemorrhages, and hard exudates are already present in the retina. Significant visual impairment occurs in the advanced stage (proliferative diabetic retinopathy (PDR)) secondary to neovascularization that causes severe bleeding and retinal detachment [3]. The primary treatment goal is to prevent further visual loss with intensive pharmacotherapeutic control of blood glucose level and management of microvascular complications using intravitreal pharmacological agents, laser photocoagulation, and vitreous surgery [3]. Therapeutics to improve residual vision, such as anti-vascular endothelial growth factor (VEGF) therapy, also gained popularity in recent years. However, their cost-effectiveness and complications such as traumatic intraocular injuries and tractional retinal detachment still outweigh the benefits. Therefore, it remains a challenge to improve residual vision in PDR patients with the available interventions.

According to the “residual vision activation theory”, strengthening of synaptic transmission and synchronization of partially damaged structures (within-systems plasticity) and downstream neuronal networks (network plasticity) is a promising alternative to reactivate and restore visual functions [4]. One way of exploring this possibility is the use of non-invasive brain stimulation (NIBS) techniques such as transcranial direct current stimulation (tDCS) and transcranial alternating current stimulation (tACS). TDCS involves the application of low-intensity (1–2 mA) direct electrical current to stimulate cortical areas through the intact head. During stimulation, tDCS modulates neuronal activity by alterations of neuronal membrane potentials caused by the opening or closing of voltage-gated ion channels [5]. As shown in the motor cortex, this effect is polarity-dependent because anodal (positive current) and cathodal (negative current) stimulation brings the resting membrane potential closer to depolarization (increase likelihood of neuronal firing) and hyperpolarization (decrease likelihood of neuronal firing), respectively [5]. The effect of tDCS on cortical excitability persists after stimulation and is attributed to prolonged synaptic efficacy changes such as long-term potentiation (LTP) and long-term depression (LTD). In general, anodal stimulation increases and cathodal stimulation decreases cortical activity [6,7].

In healthy individuals, tDCS elicited transient excitability changes in the primary visual cortex (V1) as inferred from the modulation of the N70 component of visual evoked potential (VEP) amplitude and occurrence of transcranial magnetic stimulation (TMS)-induced phosphenes [8,9,10,11]. Behaviorally, in the color discrimination task, anodal tDCS improved the blue-yellow range threshold (with no impact in the red-green range) mediated by the koniocellular pathways, while cathodal tDCS impaired discrimination within the red-green range mediated by the parvocellular pathways and at the same time increased koniocellular-driven discrimination [12]. Moreover, anodal tDCS significantly decreased cell discrimination threshold only from the most eccentric regions (peripheral) of the visual field [13]. TDCS is also beneficial for patients with visual field loss due to cortical damage. For instance, in stroke patients, tDCS improved contrast discrimination, motion and object detection, object recognition, attention or visual awareness, as well as performance in complex perceptual tasks such as face recognition and visual search [4,14]. In amblyopic patients, 15 min of anodal tDCS improved contrast sensitivity and increased VEPs of the amblyopic eye, while cathodal tDCS decreased both measures [15,16]. Cathodal tDCS over V1 contralateral to the amblyopic eye improved visual acuity and inhibited VEPs amplitude in the targeted site, while it facilitated VEPs ipsilateral to the amblyopic eye [17]. On the other hand, patients with vision loss secondary to optic neuropathies reported visual field improvement after receiving repetitive transorbital tACS. Evidence suggests that the therapeutic effect of tACS is due to re-synchronization of brain networks, which were desynchronized by vision loss [18]. However, despite the promising results, there is currently no attempt to explore the potential of tDCS as a vision rehabilitation tool for visual impairment due to retinal disease.

Retinal diseases, including the early stage of DR (NDR), are characterized by high internal noise within the visual pathways, further aggravating impaired visual functions [19,20,21]. In theory, neuronal noise can be more severe during the advanced stage of DR. For instance, resting-state functional connectivity is significantly increased between V1 and the frontal lobe in PDR patients [22]. The increase in functional connectivity can be considered compensatory but may also constitute an aberrant neural network that can interfere when a task recruits these brain regions. Therefore, we hypothesized that the hyperpolarizing effect of cathodal tDCS on neuronal membrane potentials could reduce neural noise and improve the processing of visual inputs from damaged retinas of PDR patients. We applied cathodal tDCS over V1 and measured visual acuity before and after stimulation using the Early Treatment Diabetic Retinopathy Study (ETDRS) chart. We also hypothesized that if visual acuity improves, we can observe changes in task performance associated with vision-related processes upstream from V1. For that purpose, we measured the patients’ “number acuity”, that is, the ability to discriminate the more numerous of two sets of non-verbal stimuli (e.g., dots) using a numerical discrimination task [23,24]. Number acuity is an established measure of the parietal lobe-based approximate number system (ANS) [23,25], but substantial recent evidence also suggests direct perceptual processing of numerosity in V1 [26,27,28,29,30]. The results of the present study will help us understand the impact of DR at the cortical level and develop a new vision rehabilitation tool.

## 2. Materials and Methods

### 2.1. Participants

**Figure 1:**
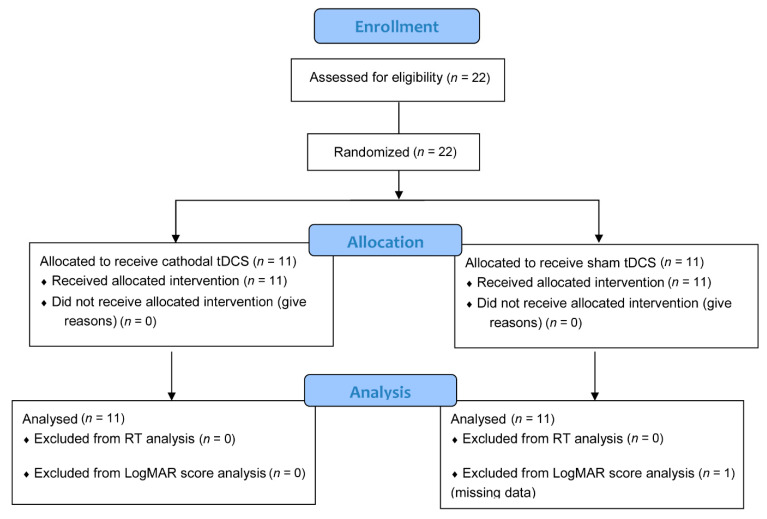
Flowchart of patient inclusion (CONSORT 2010).

Twenty-two clinically diagnosed PDR patients volunteered for the study. The number of patients surpassed our a priori calculated (G*Power 3.1.9.2) sample size (14) required to achieve a statistical power (1-β) of 90% at an alpha level of 0.05 and a moderate effect size (0.50) for a study with between-within subject design. The study was conducted on February 20, 2020, at the Nazareth General Hospital in Dagupan City, Pangasinan, Philippines. The patients are all native Filipino speakers and private patients of Dr de Venecia. They were allocated into either the tDCS group (5 females, 6 males; mean age = 57.73 years, SD = 9.45 years) or the sham group (4 females, 7 males; mean age = 52.73, SD = 12.92 years) using permuted block randomization (Figure 1). They all have corrected-to-normal vision and were right-handed except for 2 patients in the tDCS group and 1 patient in the sham group. The diabetes duration (mean ± SD) was 11.36 ± 6.32 and 13.27 ± 5.85 years for the tDCS group and sham group, respectively. Presented in Table 1 are the demographic and clinical characteristics of each patient. Except for retinopathy, there were no reports of neurological (for example, stroke and epilepsy) and psychiatric disorders (for example, depression), as well as brain injury and surgery. We also cross-checked the patients for contraindications to NIBS methods such as metallic or electrical implants in the head, neck, and chest. The experimental protocols complied with the Helsinki Declaration’s guidelines for human studies and approved by the chief of clinics representing the hospital board of Nazareth General Hospital. The study is registered at the International Standard Randomised Controlled Trial Number (ISRCTN) registry (Registration number: ISRCTN70877737). All patients signed written informed consent before the experiment.

### 2.2. Experimental Design and Procedure

The study has a double-blinded, randomized, sham-controlled design. For reliable blinding, another team member set up the stimulator, and neither the patients nor the experimenter knew the stimulation condition. We conducted the experiments in a quiet and well-lighted room. Initially, patients were brief about the study purpose and given detailed instructions (in Filipino or local dialect) to ensure they understood the task. Informed consent was acquired after all questions had been answered and thoroughly clarified. The experiment starts with the measurements of the right and left eyes’ visual acuity. Subsequently, we localized V1 following the international 10–20 electroencephalography (EEG) guidelines. The distance between the nasion and inion, as well as between the right and left pre-auricular were measured using a tape measure (cm). The two lines intersection was marked using a washable color marker and designated as the vertex. The vertex’s location served as a reference in positioning the EEG cap on the patient’s head. We used three elastic EEG caps (size 54, 56, and 58 cm) to accommodate different head sizes (EASYCAP GmbH, Germany). During the mounting of the EEG cap, the vertex always matched the location of the Cz electrode. The scalp area underlying the Oz electrode was marked and designated as the location of V1. After removing the EEG cap, the stimulating (cathode) electrode was placed on V1 and secured using an elastic rubber bandage. The reference (anode) electrode was placed on the right shoulder using adhesive medical tape. After setting up the tDCS electrodes, patients were allowed to perform practice trials. Subsequently, they performed the numerical discrimination task, which was immediately followed by the stimulation (cathodal tDCS or sham). As instructed, the patients closed their eyes and relaxed during stimulation. The patients repeated the numerical discrimination task and underwent visual acuity testing immediately after the stimulation. Each experimental session, including the preparations, lasted for 40 min.

### 2.3. Transcranial Direct Current Stimulation (tDCS)

Delivery of tDCS was done using a pair of saline-soaked (0.9%-NaCl) surface sponge electrodes connected to a battery-driven, constant-current stimulator (DC-STIMULATOR PLUS, NeuroConn Gmbh, Ilmenau, Germany). The cathode electrode was placed over V1 using the 10–20 EEG coordinates. To achieve focal stimulation of V1 and avoid the effect on other cortical areas, the reference/return (anode) electrode was placed on the right shoulder. For cathodal tDCS stimulation, the current intensity was 1 mA and was delivered continuously for 10 min. The rectangular electrodes measured 5 × 7 cm (surface area: 35 cm^2^) in diameter; therefore, the current density underneath them was 0.029 mA/cm^2^ during stimulation. The current was slowly ramped-up and ramped-down for 10 s at the start and end of stimulation, respectively. We kept the impedance during stimulation below 10 kΩ to minimize tingling skin sensation. To ensure the patients experience a similar initial skin sensation of tDCS, for sham stimulation, the current was applied for 30 s with an additional 10 s ramped-up from 0 to 1 mA, and 10 s ramped-down to 0 mA. A stimulation duration of 30 s is not known to induce after-effects [7]. All stimulation parameters conformed to the safety guidelines for tDCS [31]. After the experiment, the patients answered a standard tDCS questionnaire to document stimulation-related adverse effects.

### 2.4. Visual Acuity Assessment

A certified ophthalmologist (Dr de Venecia) conducted the visual acuity testing procedure using the standard ETDRS chart before and immediately after stimulation. The ETDRS chart has similar numbers (5) of Sloan letters per line, equal spacing between lines and letters on a log scale (0.02 logarithm of the minimum angle of resolution (LogMAR), and balanced letter difficulty in the individual lines [17]. During the test (with dimmed room light), the chart was positioned 4 m away from the patients and lit at a standard lighting level (85 cd/m^2^). Uncorrected visual acuity was measured first in the right eye, while the left eye was occluded. To prevent memorization, we used two different charts for testing the right (Chart 1) and left eye (Chart 2). The patients read the letters on the chart slowly from top to bottom, letter-by-letter, and beginning with the first letter on the top row. If a patient misread >2 letters on a line, we aborted the procedure and added 0.02 log units (for every misread letter) to the LogMAR score of that line. A high LogMAR score is indicative of worsening vision. Patients who cannot read the letters were given a LogMAR score of 1.9 for the ability to count fingers, 2.3 for detecting hand motion, 2.7 for light perception, and 3.0 for the absence of light perception [32].

### 2.5. Numerical Discrimination Task

**Figure 2:**
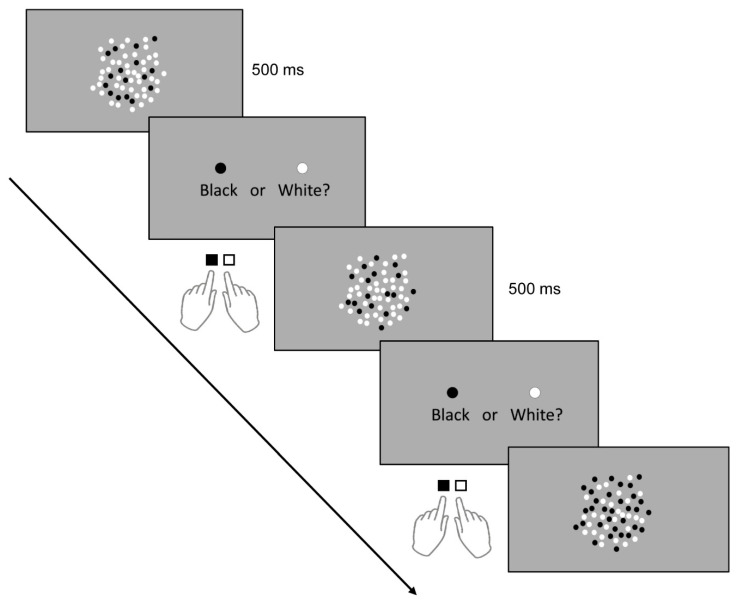
Numerical discrimination task. Stimuli (dots) were presented visually for 500 ms, and patients had to decide via button press whether there were more white or black dots.

During the task, patients sat 50 cm away from a 16-inch computer screen with a refresh rate of 60 Hz and a resolution of 1920 × 1080 (HP ENVY dv7-7388sz Notebook PC). Stimuli consisted of 60 intermixed, non-overlapping white and black dots presented on a gray background with a luminance of 36.2 cd/m² (Figure 2). The atients were instructed to indicate whether there were more black or white dots. Their left and right index fingers rested on the “B” and “N” computer keys, respectively. To familiarise the patients with task rules, they were given a short training period (10 practice trials) before the main task. The practice trials were similar to the test trials except that they only contained 40 dots. After the training, a key press starts the test trials, and the dots appeared in the middle part of the computer screen. In previous studies involving healthy individuals [23,24], the presentation time of the dots was 200 ms, which is within the known duration of rapid information processing (100–400 ms) through the visual system before response output [33]. Considering the patients’ visual impairment, we set the stimulus presentation duration to 500 ms to ensure sufficient time for stimulus processing and limit the chances of guessing. This duration, however, is insufficient for the patients to count the exact number of dots serially. The initial colors of the stimulus were yellow and blue, however, during practice trials, patients also had difficulty perceiving them; hence we replaced the color with white and black.

To isolate the effect of stimulation on numerosity, we kept other perceptual confounding stimulus variables (for example, individual dot diameter (8 mm), surface area covered by the dots (113.09 cm^2^), and inter-dot spacing or sparsity) constant across the trials [34]. Therefore, only the number (ratio) of the black and white dots changes per trial. Patients were made aware that no trial contains equal numbers of black and white dots. Once the dots disappear, an instruction appears on the screen instructing the patients to indicate their answer by pressing the “B” key for black and “N” key for white. The next trial appears after a button press. Although there was no restricted time window for responding, we instructed the patients to indicate their answers as quickly and accurately as possible. They answered two sets of 30 different test trials before and after stimulation. The appearance of test trials containing more black or more white dots was randomized. We drew the ratio of the smaller to the larger set on each trial from one of four ratio bins: 1:2, 3:4, 5:6, or 7:8 [24]. There were seven trials with a 1:2 ratio, eight trials with a 3:4 ratio, seven trials with a 5:6 ratio, and eight trials with a 7:8 ratio. The patients performed the practice and test trials binocularly without optical corrections to rule out corrective lenses’ influence in the stimulation-specific effect on residual vision. Stimulus presentation and response recording was made possible by a Psychopy-based program (Psychopy Software in Python, University of Nottingham, Nottingham, United Kingdom). The program recorded reaction time (RT) and accuracy for each trial. RT is defined as the time (seconds) from the dots’ disappearance until the patient presses a response key. The task (practice and test trials) and stimulation period were about 20 min.

### 2.6. Statistical Analysis

All data were analyzed using SPSS 26 software (IBM Corp., Armonk, NY, USA). Shapiro–Wilk and Levene’s test assessed the normality of the data distribution and homogeneity of variances, respectively. In instances of normality violation (Shapiro–Wilk test: *p* ≤ 0.05), the data are logarithmically transformed. The LogMAR scores were analyzed using a three-way (2 × 2 × 2) mixed analysis of variance (ANOVA) with a between-subject factor “stimulation” (cathodal tDCS and sham) and within-subject factors “time” (before and after stimulation) and “eye” (right and left). One patient from the sham group had missing visual acuity data; therefore, we only analyzed LogMAR scores from 11 patients in the tDCS group and 10 patients in the sham group. For the numerosity discrimination task, we calculated the average RTs before and after stimulation. Discarded RT (excluded from the analysis) are those beyond 2 standard deviations (SDs) away from the mean (outliers) and those from incorrect trials. To determine the accuracy rate (AR%), we divided the number of correct trials from the total number of trials and multiplied by 100. The RTs and ARs were analyzed using a two-way (2 × 2) mixed ANOVA with a between-subject factor “stimulation” (cathodal tDCS and sham) and a within-subject factor “time” (before and after stimulation). Effect sizes were reported as partial eta squared (η_ρ_^2^) values (small: 0.01, moderate: 0.06 and large: >0.13). If the ANOVA yielded significant results, we explored it with a Bonferroni corrected post-hoc *t*-test. In all statistical analyses, we set a threshold significance level at a *p*-value of ≤0.05. Unless otherwise stated, all reported values are mean ± SD.

## 3. Results

The tDCS and sham group have comparable mean age and diabetes duration. All patients completed the experimental procedure well. One patient in the sham group reported mild headache, neck fatigue, and increased heart rate after stimulation. He was able to go home after the symptoms alleviated. Patients in the tDCS group did not complain of any side effects. Before the stimulation, the mean LogMAR score on the right eye (sham group: 1.96 ± 0.33 log units, tDCS group: 0.99 ± 0.25 log units) significantly differed between the groups (*p* = 0.029). In contrast, the mean LogMAR score on the left eye (sham group: 1.04 ± 0.24 log units, tDCS group: 1.25 ± 0.26 log units) was comparable between the groups (*p* = 0.835). The numerosity discrimination performance was comparable between the groups as indicated by the mean RT (sham group: 1.22 ± 0.20 s, tDCS group: 2.47 ± 0.53 s) (*p* = 0.056) and mean AR (sham group: 74.24 ± 3.65%, tDCS group: 74.24 ± 4.94%) (*p* = 0.898) before the stimulation (Table S1).

### 3.1. Visual Acuity

**Figure 3:**
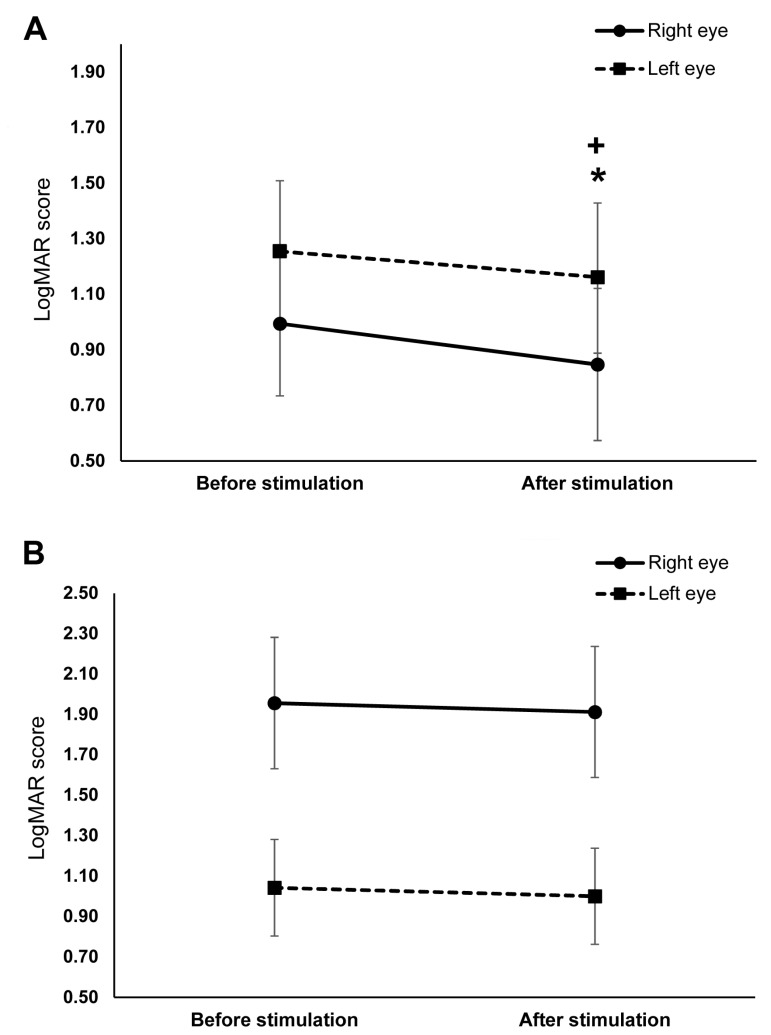
The effects of cathodal transcranial direct current stimulation (tDCS) and sham stimulation on visual acuity. The *y*-axis displays the mean LogMAR scores. The *x*-axis displays the time points of the LogMAR score measurement. (**A**) Visual acuity before and after cathodal tDCS stimulation. The right (straight line) and left (dashed line) eye’s LogMAR scores significantly decreased after stimulation. (**B**) Visual acuity before and after sham stimulation. There were no significant changes in the LogMAR scores of both eyes after sham stimulation. + = significant differences between the pre- and post-stimulation LogMAR score of the right eye (*p* ≤ 0.05), * = significant differences between the pre- and post-stimulation LogMAR score of the left eye (*p* ≤ 0.05). Presented data are mean values ± standard error of the mean (SEM).

The data used for the final analysis was normally distributed after logarithmic transformation and have equal group variances (all *p* > 0.05). The ANOVA conducted on the log-transformed LogMAR scores revealed a significant main effect of time (*F* (1,19) = 14.97, *p* = 0.001, η_ρ_^2^ = 0.441) such that the overall post-stimulation score (1.15 ± 0.82) was significantly lower than before stimulation (1.42 ± 0.94). The eye and stimulation interaction was significant (*F* (1,19) = 4.83, *p* = 0.041, η_ρ_^2^ = 0.203). Bonferroni corrected post hoc comparisons indicate that the interaction was mainly driven by the significantly lower overall score of the right eye in the tDCS group (0.99 ± 0.84) than the right eye in the sham group (1.96 ± 0.33) (*p* = 0.016) (Figure 3A,B). In contrast, the overall score of the left eye in the tDCS group (1.25 ± 0.26) and sham group (1.04 ± 0.24) were comparable (*p* = 0.965). 

**Figure 4:**
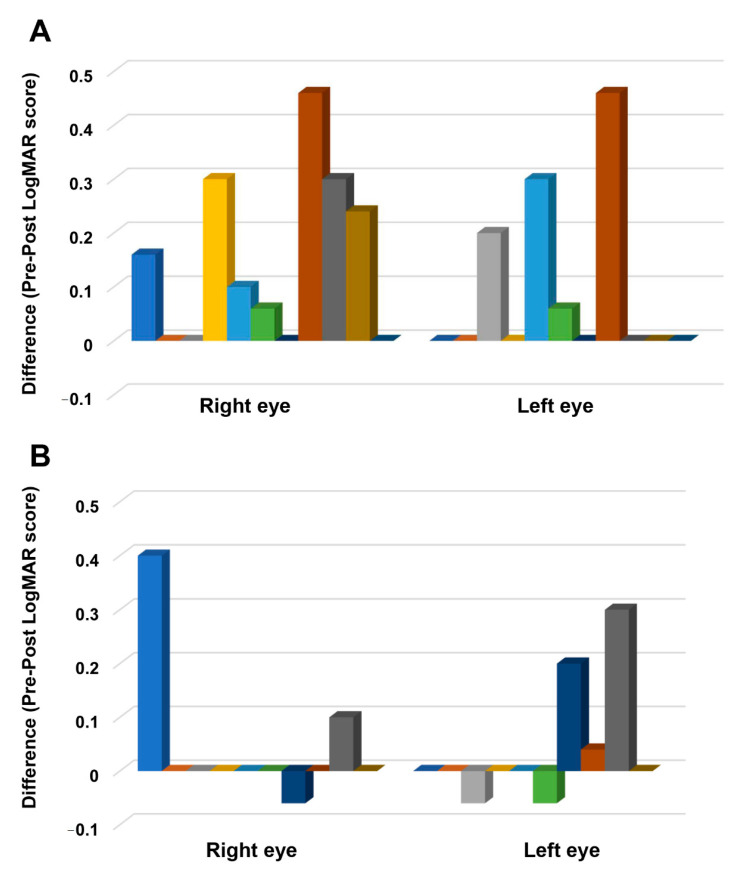
The difference between pre- and post-stimulation LogMAR scores. The *y*-axis displays the difference between the before and after stimulation LogMAR scores. The *x*-axis indicates the eye where the measurements are conducted. Each column represents the individual participant’s score in the tDCS (**A**) and sham group (**B**). A positive score indicates improvement, and a negative score suggests worsening of visual acuity after stimulation.

The time and stimulation interaction was also significant (*F* (1,19) = 8.92, *p* = 0.008, η_ρ_^2^ = 0.319). Bonferroni corrected post hoc comparisons showed that the overall score in the tDCS group (before stimulation: 1.13 ± 0.94, after stimulation: 1.00 ± 0.95) significantly decreased after stimulation (*p* ≤ 0.001), whereas the decrease in the overall score in the sham group (before stimulation: 1.49 ± 0.98, after stimulation: 1.45 ± 0.99) was not significant (*p* = 0.549). The three-way interactions of stimulation, time, and eye was not significant (Table 2). Planned exploratory post hoc comparisons revealed that this result was mainly driven by the absence of significant differences in the sham group’s pre- and post-stimulation scores of the right (before stimulation: 1.96 ± 0.33, after stimulation: 1.91 ± 0.32; *p* = 0.794) and left eye (before stimulation: 1.04 ± 0.24, after stimulation: 1.00 ± 0.24; *p* = 0.542) (Figure 3B); and second, the absence of significant differences in the left eye’s pre-stimulation (tDCS: 1.25 ± 0.86, sham: 1.04 ± 0.75; *p* = 0.835) and post-stimulation scores (tDCS: 1.16 ± 0.90, sham: 1.00 ± 0.75; *p* = 0.909) between stimulation conditions. However, post hoc comparisons in the tDCS group revealed a significant decrease in the right (before stimulation: 0.99 ± 0.25, after stimulation: 0.85 ± 0.27; *p* = 0.001) and left eye’s scores (before stimulation: 1.25 ± 0.26, after stimulation: 1.16 ± 0.27; *p* = 0.020) (Figure 3A). The right eye’s post-stimulation scores also differed significantly between stimulation conditions, with the score in the tDCS group (0.85 ± 0.88) being lower than in the sham group (1.91 ± 1.03) (*p* = 0.011). Figure 4 shows the difference between the pre- and post-stimulation LogMAR scores of individual patients in the tDCS and sham group.

### 3.2. Number Acuity

**Figure 5:**
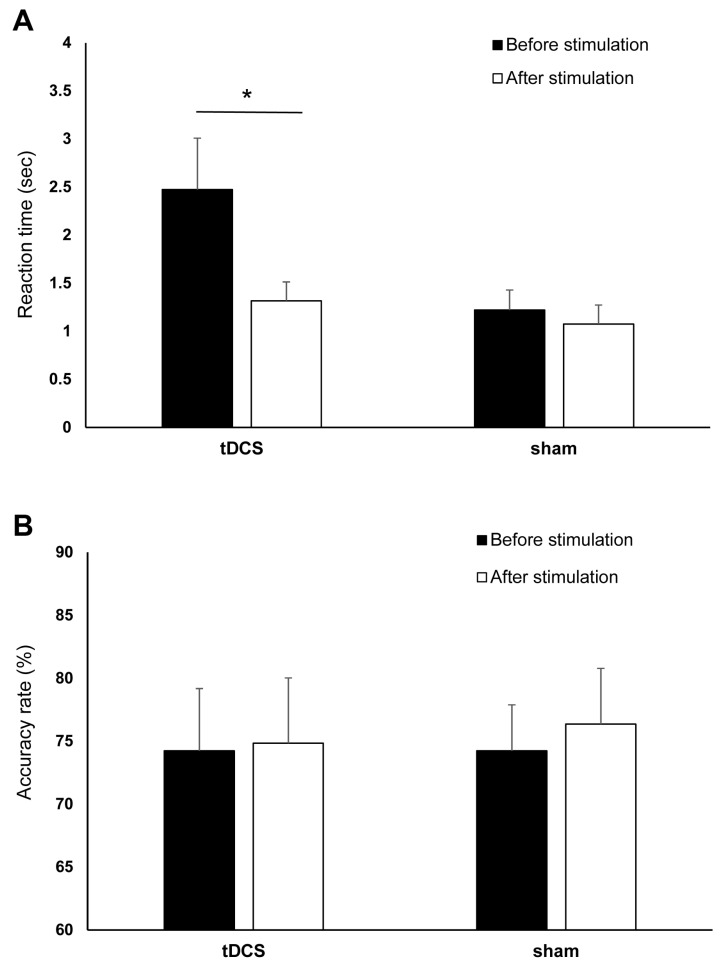
The effects of cathodal tDCS and sham stimulation on numerical discrimination. (**A**) Reaction time (RT) before and after cathodal tDCS and sham stimulation. The *y*-axis displays the mean RT (in seconds). The *x*-axis displays the time points of the RTs measurements in the tDCS and sham groups. RT significantly decreased after cathodal tDCS but not after sham. (**B**) Accuracy rate (AR) before and after cathodal tDCS and sham stimulation. The *y*-axis displays the mean AR (%). The *x*-axis displays the time points of the error measurement in the tDCS and sham group. There were no significant changes in AR after tDCS and sham stimulation. tDCS = transcranial direct current stimulation, RT = reaction time, AR = accuracy rate, * = Significant differences between the pre- and post-stimulation measurements (*p* ≤ 0.05). Presented data are mean values±standard error of the mean (SEM).

**Figure 6:**
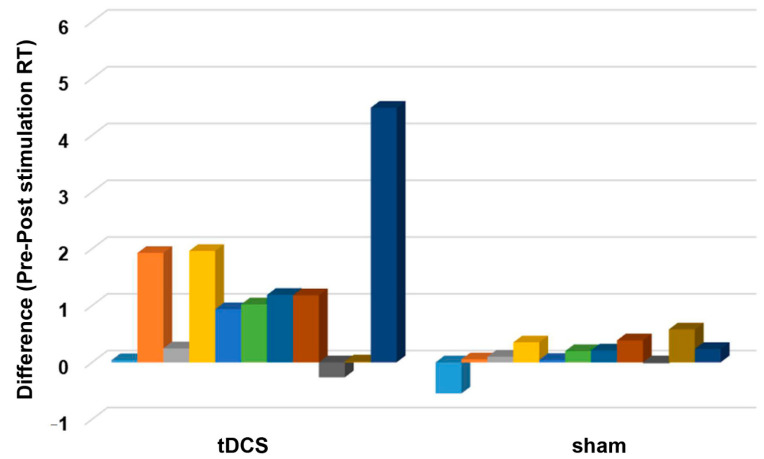
The difference between pre- and post-stimulation reaction time (RT). The *y*-axis displays the difference between the before and after stimulation reaction time (s). The *x*-axis indicates the groups (tDCS and sham). Each column represents the individual participant’s reaction time difference. A positive and negative score indicates a shorter and longer reaction time after stimulation, respectively.

For the RTs, the final dataset included 976 trials (73.94% of 1320 trials). RTs from incorrect trials (331) and outliers (13) accounted for 25.08% and 0.98% of the full dataset, respectively, and were excluded in the final analysis. The ANOVA for the log-transformed RTs yielded a significant main effect of time (*F* (1,20) = 22.02, *p* ≤ 0.001, η_ρ_^2^ = 0.524) as indicated by the shorter post-stimulation overall RT (before stimulation: 1.85 ± 1.03 s, after stimulation: 1.19 ± 0.05 s). The main effect of stimulation was not significant (*F* (1,20) = 2.665, *p* = 0.118, η_ρ_^2^ = 0.118), indicating comparable overall RT between tDCS (1.89 ± 1.33 s) and sham (1.15 ± 1.33 s) group. However, the effect of time and stimulation interaction was significant (*F* (1,20) = 5.85, *p* = 0.025, η_ρ_^2^ = 0.226). Bonferroni corrected post hoc comparisons showed that RTs significantly decreased in the tDCS group (before stimulation: 2.47 ± 1.77 s, after stimulation: 1.32 ± 0.65 s) (*p* ≤ 0.001). However, the RTs were comparable before (1.22 ± 0.68 s) and after (1.07 ± 0.65 s) sham stimulation (*p* = 0.124) (Figure 5A). Figure 6 shows the difference between the pre- and post-stimulation RT of individual patients in the tDCS and sham group. The ANOVA for the AR yielded no significant results (Table 2, Figure 5B).

## 4. Discussion

The present study explored the effect of tDCS stimulation in PDR patients’ residual vision. The mean LogMAR score of both eyes and mean RT in numerical discrimination task significantly improved in patients who underwent cathodal tDCS stimulation of V1. Diabetes duration is comparable between the groups and may not have influenced the stimulation’s effectiveness (for example, stimulation is less effective for patients with more chronic diabetes). Therefore, the results indicate that cathodal tDCS stimulation of V1 improves the patients’ visual and number acuity.

### 4.1. The Effect of Cathodal tDCS on Visual Acuity

The cathodal tDCS-induced improvement in visual acuity is robust as indicated by the large effect sizes observed on significant interactions involving the factor stimulation (all η_ρ_^2^ > 0.13). These results are consistent with our a priori hypothesis that decreasing resting membrane potentials and spontaneous neuronal firing rates in V1 with cathodal tDCS can improve the patients’ vision. Concerning the underlying mechanisms, we argue based on the evidence that high internal noise within the visual pathways contributes to vision impairment in retinal diseases [19,20,21]. Studies suggest that noise-processing neurons act more randomly than neurons that process functional signals; therefore, the inhibitory effect of cathodal tDCS would be more robust over the noise and increase the signal-to-noise ratio in the neuronal network [14,35]. The increased signal-to-noise ratio may boost neural computations in V1 needed for efficient perception and interpretation of sensory signals from eyes with compromised retinal functions. In contrast, modulation of neuronal signals against background noise may not have occurred in the sham group since their visual acuity did not improve after stimulation. Our assumption is in accordance with the findings in amblyopic patients who receive cathodal tDCS over V1 contralateral to the affected eye. In these patients, in addition to a possible stimulation-induced decrease in transcallosal inhibition, a reduction in V1 neuronal excitability as indicated by the reduction of VEPs amplitude may have facilitated visual acuity improvement [17]. Interestingly, cathodal tDCS impaired Vernier acuity, while anodal tDCS improved Vernier and visual acuity (measured with a Landolt gap task) in healthy young individuals [36,37]. Anodal tDCS of V1 also increased VEPs amplitude and improved contrast sensitivity in a group of amblyopic patients who were relatively younger than the participants in the Bocci et al. study [16]. These results theoretically suggest that in cases where neuronal noise is less robust such as in an intact visual system or in patients with less chronic visual system pathology, anodal tDCS may enhance visual acuity by boosting functional neuronal signals. On the other hand, cathodal tDCS can be detrimental in such cases because it may impair both noise and functional neuronal signals. In contrast, in more chronic or advanced visual system disorders, where neuronal noise is more robust, cathodal tDCS can be more beneficial because of its inhibitory effect.

### 4.2. The Effect of Cathodal tDCS on Number Acuity

The decrease in RT after cathodal tDCS was also robust, as indicated by the large effect size of the time and stimulation interaction (η_ρ_^2^ = 0.226). In contrast, cathodal tDCS had a limited impact on accuracy because the analysis of ARs revealed no significant results. This is not surprising because task difficultly is low, and the patients’ ARs are already high (mean AR: 74.24%) before stimulation. Therefore, the patients could have immediately reached a ceiling effect on task performance. Nonetheless, there is no indication of speed-accuracy trade-off (decrease in RT with an increase AR or vice versa) in either the cathodal tDCS or sham group. Overall, although the between groups’ AR is comparable, patients who received cathodal tDCS were faster in discriminating whether there were more black or more white dots than those who received sham stimulation. Here, we argue that this is due to the modulation of the underlying neural mechanism behind the perceptual processing of numerosity, such as discrimination and encoding (individuation of dots) in V1 by cathodal tDCS [27,28,29,30,38]. The hyperpolarizing effect of cathodal tDCS may tune or denoise the activity of V1 neurons, particularly those with receptive fields that respond best to outline, contour, and edges defined by the ratio-dependent distribution of black and white dots in space [39,40,41]. By serving as a “noise filter” in the early visual areas, improvement in visuospatial information processing in other regions of the ventral and dorsal visual streams such as the temporal (object processing), parietal (spatial processing), and frontal (decision making) cortices can be considered a secondary effect of cathodal tDCS stimulation of V1 [25,42]. V1 cathodal stimulation may diminish the robust resting-state functional connectivity between V1 and the frontal lobe, which can interfere with the frontal eye-fields’ critical function for saccadic eye movements and perceptual decision-making [22,43]. Furthermore, tDCS may have modulated the ratio-dependent number processing system that responds selectively to nonsymbolic quantities (e.g., dot arrays) of larger ratios (3:1 or 4:1) reported in the subcortical monocular portion of V1 [26]. Interestingly, there are no significant differences in AR or RT between ratio conditions in our study, indicating the absence of cathodal tDCS inhibitory effect on visual attentional skills on which numerosity perception depends. One possible explanation is that our task specifically recruits the ANS, which is relatively “attentional-free” compared to the attentional dependent “subitizing system” (for low numerosities: <4 dots) and the attentional demanding “texture-density mechanism” operating for high dense/numerous stimuli (>60 dots) [44,45] This is because although the ratio of black and white dots is allowed to vary in our task, the total number of dots in the array is fixed (60) as well as other features such as dots sparsity. Modulation of the subcortical monocular portion of V1 with tDCS is possible trans-synaptically [46]. However, this assumption remains to be determined in PDR patients using NIBS techniques with deeper penetration, such as TMS.

### 4.3. Limitations

In the present study, there were some limitations that we should address. First, contrast sensitivity (CS) is not measured because of the unavailability of a standard CS test. We considered this a significant limitation because there is evidence indicating that CS measurement can provide better information about the impact of intrinsic noise in early-stage diabetic patients [21]. In the case of patients with PDR, the effect of neuronal noise is not yet clearly understood. Further investigations should address the effects of tDCS on CS in NDR and PDR patients. Second, for safety reason, the patients’ maintenance medications (Table 1) were not discontinued during the experiment. We cannot entirely rule out the influence of these medications on the effect of tDCS, particularly those that can pass the blood-brain-barrier. For future studies, discontinuation of drugs may be possible at least 24 h before the stimulation in patients with stable blood glucose level and those taking fewer medications. Third, we did not identify the dominant eye of each patient. Although the significance of eye dominance is yet to be established [47], particularly in the field of brain stimulation, it is tempting to assume that tDCS effects may differ on the dominant and non-dominant eyes, because in theory, the dominant one provides more input to the visual cortex. Fourth, the patients did not receive anodal stimulation, and therefore the polarity-dependent effect of tDCS is not tested in the present study. Future experiments must apply anodal, cathodal, and sham stimulation in a within-subject design to systematically control for stimulation-specific effects. Finally, our sample size is relatively small, and extrapolating the results to all diabetic patients must be cautioned. Additional studies with a larger sample size are needed further to explore the impact of tDCS in V1 of diabetic patients.

## 5. Conclusions

The present study provides preliminary evidence that non-invasive brain stimulation methods such as tDCS can improve PDR patients’ residual vision. The results demonstrated the promising potential of tDCS as a vision rehabilitation tool for patients with other retinal diseases.

## Figures and Tables

**Table 1:** Demographic and clinical characteristics of the study population.

	Gender	Age	DM Duration (Years)	Comorbid Conditions	Medications
tDCS group					
1	M	42	18	Diabetic nephropathy	Insulin
2	F	65	10	Diabetic nephropathy	Insulin
3	F	54	5		Insulin, Gliclazide, Keto-analogs + essential amino acids
4	M	60	5	Hypertension	Metformin, Simvastatin, Amlodipine
5	F	71	10	Hypertension	Gliclazide, Pioglitazone, Losartan, Rosuvastatin
6	M	56	22		Metformin, Pioglitazone
7	F	59	8	Hypertension	Insulin, Linagliptin, Aspirin, Amlodipine, Losartan
8	M	69	5	Hypertension	Metformin, Losartan
9	F	63	15	Hypertension	Metformin, Gliclazide, Amlodipine, Vitamins B-complex
10	M	53	7	Cardiac disease	Gliclazide, Aspirin, Losartan, Amlodipine
11	M	43	20	Hypertension	Metformin, Amlodipine, Ferrous sulfate
Sham group					
1	M	35	14		Metformin, Atorvastatin
2	M	41	15		Insulin
3	M	48	25	Hypertension, asthma, diabetic nephropathy	Amlodipine, Valsartan, Calcium carbonate, Ferrous sulfate + Vitamin B-complex + folate
4	F	59	10		Metformin, Aspirin, Simvastatin
5	M	63	5	Prostatic hyperplasia, hypertension	Telmisartan, Glimepiride, Finasteride, Keto-analogs + essential amino acids
6	F	59	15	Hypertension	Insulin, Empagliflozin
7	M	46	10		Metformin
8	F	35	10	Cardiac disease, hypertension	Furosemide, Insulin, Warfarin, Carvedilol, Valsartan, Teneligliptin, Spironolactone, Rosuvastatin, Vitamin B12, Linagliptin, Keto-analogs
9	M	76	22	Hypertension	Losartan, Amlodipine, Insulin
10	M	55	10	Hypertension	Metformin
11	F	63	10		Insulin

DM, diabetes mellitus; PDR, proliferative diabetic retinopathy; tDCS, transcranial direct current stimulation; M, male; F, female.

**Table 2:** Results of the analyses of variances (ANOVAs) performed on the logarithm of the minimum angle of resolution (LogMAR) score and accuracy rate.

	Numerator *df*	Denominator *df*	*F*-Value	*p*-Value	η_ρ_^2^
LogMAR score					
Stimulation	1	19	2.19	0.104	0.133
Time	1	19	14.97	0.001 *	0.441
Eye	1	19	0.51	0.482	0.026
Time × stimulation	1	19	8.92	0.008 *	0.319
Time × eye	1	19	1.13	0.301	0.056
Eye × stimulation	1	19	4.83	0.041 *	0.203
Stimulation × time × eye	1	19	1.56	0.227	0.076
Accuracy rate					
Stimulation	1	20	0.53	0.821	0.003
Time	1	20	0.18	0.677	0.009
Time × stimulation	1	20	0.12	0.732	0.006

* = *p* < 0.05, *df* = Degrees of freedom.

## Data Availability

Data is contained within the article or supplementary material.

## Supplementary Materials

The following are available online at https://www.mdpi.com/2076-3425/11/2/270/s1, Table S1: LogMAR scores, mean reaction times and mean accuracy rates of individual patient before and after stimulation.

## References

[1] Wild S., Roglic G., Green A., Sicree R., King H. (2004). Global Prevalence of Diabetes. Diabetes Care..

[2] Mansour S.E., Browning D.J., Wong K., Flynn H.W., Bhavsar A.R. (2020). The Evolving Treatment of Diabetic Retinopathy. Clin. Ophthalmol..

[3] Wang W., Lo A.C.Y. (2018). Diabetic Retinopathy: Pathophysiology and Treatments. Int. J. Mol. Sci..

[4] Sabel B.A., Thut G., Haueisen J., Henrich-Noack P., Herrmann C.S., Hunold A., Kammer T., Matteo B., Sergeeva E.G., Waleszczyk W. (2020). Vision modulation, plasticity and restoration using non-invasive brain stimulation—An IFCN-sponsored review. Clin. Neurophysiol..

[5] Stagg C.J., Nitsche M.A. (2011). Physiological Basis of Transcranial Direct Current Stimulation. Neuroscience.

[6] Nitsche M.A., Cohen L.G., Wassermann E.M., Priori A., Lang N., Antal A., Paulus W., Hummel F., Boggio P.S., Fregni F. (2008). Transcranial direct current stimulation: State of the art 2008. Brain Stimul..

[7] Nitsche M.A., Paulus W. (2000). Excitability changes induced in the human motor cortex by weak transcranial direct current stimulation. J. Physiol..

[8] Antal A., Kincses T.Z., Nitsche M.A., Paulus W. (2003). Manipulation of phosphene thresholds by transcranial direct current stimulation in man. Exp. Brain Res..

[9] Antal A., Kincses T.Z., Nitsche M.A., Paulus W. (2003). Modulation of moving phosphene thresholds by transcranial direct current stimulation of V1 in human. Neuropsychologia.

[10] Antal A., Kincses Z.T., Nitsche M., Bartfai O., Paulus W. (2004). Excitability changes induced in the human primary visual cortex by transcranial direct current stimulation: Direct electrophyiological evidence. Investig. Ophthalmol. Vis. Sci..

[11] Wunder S., Hunold A., Fiedler P., Schlegelmilch F., Schellhorn K., Haueisen J. (2018). Novel bifunctional cap for simultaneous electroencephalography and transcranial electrical stimulation. Sci. Rep..

[12] Costa T.L., Nagy B.V., Barboni M.T.S., Boggio P.S., Ventura D.F. (2012). Transcranial direct current stimulation modulates human color discrimination in a pathway-specific manner. Front. Psychiatry.

[13] Costa T.L., Gualtieri M., Barboni M.T.S., Katayama R.K., Boggio P.S., Ventura D.F. (2015). Contrasting effects of transcranial direct current stimulation on central and peripheral visual fields. Exp. Brain Res..

[14] Costa T.L., Lapenta O.M., Boggio P.S., Ventura D.F. (2015). Transcranial direct current stimulation as a tool in the study of sensory-perceptual processing. Atten. Percept. Psychophys..

[15] Spiegel D.P., Byblow W.D., Hess R.F., Thompson B. (2013). Anodal Transcranial Direct Current Stimulation Transiently Improves Contrast Sensitivity and Normalises Visual Cortex Activation in Individuals With Amblyopia. Neurorehabil. Neural Repair.

[16] Ding Z., Li J., Spiegel D.P., Chen Z., Chan L., Luo G., Yuan J., Deng D., Yu M., Thompson B. (2016). The effect of transcranial direct current stimulation on contrast sensitivity and visual evoked potential amplitude in adults with amblyopia. Sci. Rep..

[17] Bocci T., Nasini F., Caleo M., Restani L., Barloscio D., Ardolino G., Priori A., Maffei L., Nardi M., Sartucci F. (2018). Unilateral Application of Cathodal tDCS Reduces Transcallosal Inhibition and Improves Visual Acuity in Amblyopic Patients. Front. Behav. Neurosci..

[18] Gall C., Schmidt S., Schittkowski M.P., Antal A., Ambrus G.G., Paulus W., Dannhauer M., Michalik R., Mante A., Bola M. (2016). Alternating Current Stimulation for Vision Restoration after Optic Nerve Damage: A Randomised Clinical Trial. PLoS ONE.

[19] Pelli D.G., Levi D.M., Chung S.T.L. (2004). Using visual noise to characterize amblyopic letter identification. J. Vis..

[20] McAnany J.J., Alexander K.R., Genead M.A., Fishman G.A. (2013). Equivalent Intrinsic Noise, Sampling Efficiency, and Contrast Sensitivity in Patients With Retinitis Pigmentosa. Investig. Opthalmol. Vis. Sci..

[21] McAnany J.J., Park J.C. (2018). Reduced Contrast Sensitivity is Associated With Elevated Equivalent Intrinsic Noise in Type 2 Diabetics Who Have Mild or No Retinopathy. Investig. Opthalmol. Vis. Sci..

[22] Yu Y., Lan D.-Y., Tang L.-Y., Su T., Li B., Jiang N., Liang R.-B., Ge Q.-M., Li Q.-Y., Shao Y. (2020). Intrinsic functional connectivity alterations of the primary visual cortex in patients with proliferative diabetic retinopathy: A seed-based resting-state fMRI study. Ther. Adv. Endocrinol. Metab..

[23] Cappelletti M., Gessaroli E., Hithersay R., Mitolo M., Didino D., Kanai R., Kadosh R.C., Walsh V. (2013). Transfer of Cognitive Training across Magnitude Dimensions Achieved with Concurrent Brain Stimulation of the Parietal Lobe. J. Neurosci..

[24] Halberda J., Mazzocco M.M.M., Feigenson L. (2008). Individual differences in non-verbal number acuity correlate with maths achievement. Nature.

[25] Dehaene S., Piazza M., Pinel P., Cohen L. (2003). Three Parietal Circuits for Number Processing. Cogn. Neuropsychol..

[26] Collins E., Park J., Behrmann M. (2017). Numerosity representation is encoded in human subcortex. Proc. Natl. Acad. Sci. USA.

[27] Fornaciai M., Brannon E.M., Woldorff M.G., Park J. (2017). Numerosity processing in early visual cortex. Neuroimage.

[28] Guillaume M., Mejias S., Rossion B., Dzhelyova M., Schiltz C. (2018). A rapid, objective and implicit measure of visual quantity discrimination. Neuropsychologia.

[29] DeWind N.K., Park J., Woldorff M.G., Brannon E.M. (2019). Numerical encoding in early visual cortex. Cortex.

[30] van Rinsveld A., Guillaume M., Kohler P.J., Schiltz C., Gevers W., Content A. (2020). The neural signature of numerosity by separating numerical and continuous magnitude extraction in visual cortex with frequency-tagged EEG. Proc. Natl. Acad. Sci. USA.

[31] Matsumoto H., Ugawa Y. (2017). Adverse events of tDCS and tACS: A review. Clin. Neurophysiol. Pract..

[32] Lange C., Feltgen N., Junker B., Schulze-Bonsel K., Bach M. (2009). Resolving the clinical acuity categories “hand motion” and “counting fingers” using the Freiburg Visual Acuity Test (FrACT). Graefe’s Arch. Clin. Exp. Ophthalmol..

[33] Foxe J., Simpson G. (2002). Flow of activation from V1 to frontal cortex in humans. Exp. Brain Res..

[34] Reinhart R.M.G., Zhu J., Park S., Woodman G.F. (2015). Medial–Frontal Stimulation Enhances Learning in Schizophrenia by Restoring Prediction Error Signaling. J. Neurosci..

[35] Antal A., Paulus W. (2008). Transcranial Direct Current Stimulation and Visual Perception. Perception.

[36] Reinhart R.M.G., Xiao W., McClenahan L.J., Woodman G.F. (2016). Electrical Stimulation of Visual Cortex Can Immediately Improve Spatial Vision. Curr. Biol..

[37] Bonder T., Gopher D., Yeshurun Y. (2018). The Joint Effects of Spatial Cueing and Transcranial Direct Current Stimulation on Visual Acuity. Front. Psychol..

[38] Salthouse T.A., Birren J.E., Schaie K.W. (1985). Speed of behavior and its implications for cognition. Handbook of the Psychology of Aging.

[39] Koulakov A., Chklovskii D. (2001). Orientation Preference Patterns in Mammalian Visual Cortex: A Wire Length Minimisation Approach. Neuron.

[40] Sasaki Y., Watanabe T. (2004). The primary visual cortex fills in color. Proc. Natl. Acad. Sci. USA.

[41] Seymour K., Clifford C.W.G., Logothetis N.K., Bartels A. (2009). Coding and Binding of Color and Form in Visual Cortex. Cereb. Cortex..

[42] Chan J.S., Newell F.N. (2008). Behavioral evidence for task-dependent “what” versus “where” processing within and across modalities. Percept. Psychophys..

[43] Murd C., Moisa M., Grueschow M., Polania R., Ruff C.C. (2020). Causal contributions of human frontal eye fields to distinct aspects of decision formation. Sci. Rep..

[44] Anobile G., Tomaiuolo F., Campana S., Cicchini G.M. (2020). Three-systems for visual numerosity: A single case study. Neuropsychologia.

[45] Pomè A., Anobile G., Cicchini G.M., Scabia A., Burr D.C. (2019). Higher attentional costs for numerosity estimation at high densities. Atten. Percept. Psychophys..

[46] Nonnekes J., Arrogi A., Munneke M.A.M., van Asseldonk E.H.F., Nijhuis L.B.O., Geurts A.C., Weerdesteyn V. (2014). Subcortical Structures in Humans Can Be Facilitated by Transcranial Direct Current Stimulation. PLoS ONE.

[47] AMapp P., Ono H., Barbeito R. (2003). What does the dominant eye dominate? A brief and somewhat contentious review. Percept. Psychophys..

