# Supplementary material for: Visual Cortex Transcranial Direct Current Stimulation for Proliferative Diabetic Retinopathy Patients: A Double-Blinded Randomized Exploratory Trial
Source: Brain Sci. 2021 Feb 21;11(2):270. doi: 10.3390/brainsci11020270 (PMC7924823; doi:10.3390/brainsci11020270)
Supplement: Supplementary file 1 [file brainsci-11-00270-s001.pdf]

Supplementary Materials

Table S1. LogMAR scores, mean reaction times and mean accuracy rates of individual patient before and after stimulation.

	**LogMAR score before stimulation (log unit)**		**LogMAR score after stimulation (log unit)**		**RT before stimulation (sec)**	**RT after stimulation (sec)**	**AR before stimulation (%)**	**AR after stimulation (%)**
**Sham**	**Right eye**	**Left eye**	**Right eye**	**Left eye**				
1	2.30	3.00	1.90	3.00	1.200930426	1.746164861	66.66	56.66
2	3.00	0.60	3.00	0.60	0.479694355	0.428108942	86.66	96.66
3	1.00	0.40	1.00	0.46	0.744769603	0.646360788	73.33	83.33
4	3.00	1.30	3.00	1.30	1.620026137	1.269536318	83.33	80
5	2.70	0.70	2.70	0.70	0.317538964	0.274474409	90	90
6	0.36	1.02	0.36	1.08	0.699528571	0.502518114	66.66	70
7					0.597526901	0.388824886	76.66	76.66
8	0.90	0.90	0.96	0.70	1.535388639	1.148447791	63.33	70
9	3.00	0.70	3.00	0.66	1.930688555	1.947173265	50	46.66
10	1.00	1.30	0.90	1.00	2.15674003	1.577645105	73.33	86.66
11	2.30	0.50	2.30	0.50	2.151882079	1.9172486	86.66	83.33
12	2.30	3.00	1.90	3.00	1.200930426	1.746164861	66.66	56.66
**tDCS**								
1	0.46	1.90	0.30	1.90	0.623135621	0.584139039	66.66	66.66
2	0.90	1.90	0.90	1.90	4.233880479	2.30780711	76.66	80
3	1.90	0.66	1.90	0.46	0.820385531	0.575180381	63.33	76.66
4	0.50	2.30	0.20	2.30	3.422959293	1.461458293	50	36.66
5	0.40	0.60	0.30	0.30	1.896772719	0.961491378	86.66	96.66
6	0.36	0.38	0.30	0.32	2.077262805	1.056846863	96.66	90
7	0.40	1.30	0.40	1.30	2.283729186	1.096498092	70	86.66
8	1.36	1.46	0.90	1.00	3.490134569	2.310843279	83.33	80
9	1.30	0.40	1.00	0.40	1.377072078	1.63864993	86.66	83.33
10	0.36	0.20	0.12	0.20	0.623907453	0.609495288	90	73.33
11	3.00	2.70	3.00	2.70	6.37358256	1.889200617	46.66	53.33
12	0.46	1.90	0.30	1.90	0.623135621	0.584139039	66.66	66.66

LogMAR, Logarithm of the Minimum Angle of Resolution; tDCS, Transcranial direct current stimulation; RT, Reaction time; AR, Accuracy rate.
